# Minimally displaced unilateral facet fracture of cervical spine can lead to spinal cord injury: a report of two cases

**DOI:** 10.1186/s12891-021-04025-x

**Published:** 2021-02-11

**Authors:** Satoshi Maki, Mitsuhiro Kitamura, Takeo Furuya, Takuya Miyamoto, Sho Okimatsu, Yasuhiro Shiga, Kazuhide Inage, Sumihisa Orita, Yawara Eguchi, Seiji Ohtori

**Affiliations:** Department of Orthopaedic Surgery, Chiba University Graduate School of Medicine, 1-8-1 Inohana, Chuou-Ku, Chiba, 260-8670 Japan

**Keywords:** Unilateral minimally displaced facet fractures, Spinal injury, Spinal cord injury, Vertebral artery injury

## Abstract

**Background:**

According to most of the commonly used classification systems for subaxial spine injuries, unilateral and minimally displaced facet fractures without any sign of a spinal cord injury would be directed to non-operative management. However, the failure rate of non-operative treatment varies from 20 to 80%, and no consensus exists with regard to predictors of failure after non-operative management.

**Case presentation:**

Case 1 is a patient with a unilateral facet fracture. The patient had only numbness in the right C6 dermatome but failed non-operative treatment, which resulted in severe spinal cord injury. Case 2 is a patient who had a similar injury pattern as case 1 but presented with immediate instability and underwent fusion surgery. Both patients had a minimally displaced unilateral facet fracture accompanied by disc injury and blunt vertebral artery injury, which are possible signs indicating significant instability.

**Conclusions:**

This is the first report of an isolated unilateral facet fracture that resulted in catastrophic spinal cord injury. These two cases illustrate that an isolated minimally displaced unilateral facet fracture with disc injury and vertebral artery injury were associated with significant instability that can lead to spinal cord injury.

## Background

Isolated minimally displaced facet fractures occur in 5% of all traumatic cervical ﻿spine injuries [1]. According to most of the commonly used classification systems for subaxial spine injuries [2, 3], unilateral and minimally displaced facet fractures without any sign of a spinal cord injury would be directed to non-operative management. However, the failure rate of non-operative treatment varies from 20 to 80%, and no consensus exists with regard to predictors of failure after non-operative management [4–6]. The first patient with an unilateral facet fracture had only numbness in the right C6 dermatome but failed non-operative treatment, resulting in severe spinal cord injury. The second patient presented with a similar injury pattern as the first one. Both involved an unilateral facet fracture, disc injury and blunt vertebral artery injury (VAI), which are possible signs indicating significant instability. However, the second patient presented with central cord syndrome and obvious instability immediately after injury and underwent surgical stabilization. To the best of our knowledge, this is the first report in which non-operative treatment of unilateral, minimally displaced facet fracture resulted in catastrophic spinal cord injury.

## Case presentation

### Case 1

**Fig. 1:**
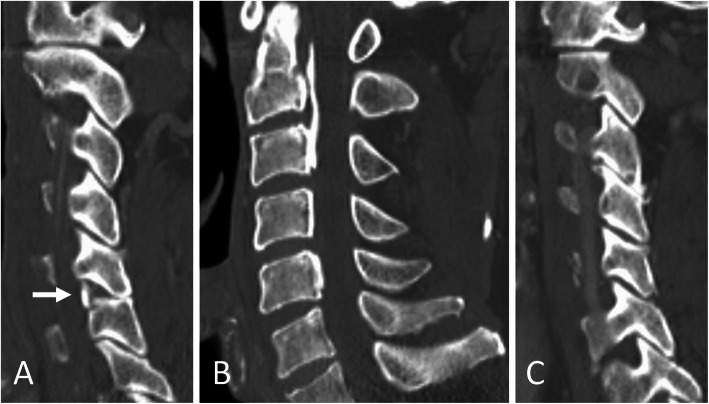
**a** Right parasagittal **b** midsagittal and **c** left parasagittal computed tomography scan of the cervical spine showed a right minimally displaced facet fracture of C6 (arrow). Small ossifications of the longitudinal ligament were seen at C2–3 and C5, and the canal was narrow. There were no signs of subluxation of the left facet joint or disc widening in these images

**Fig. 2:**
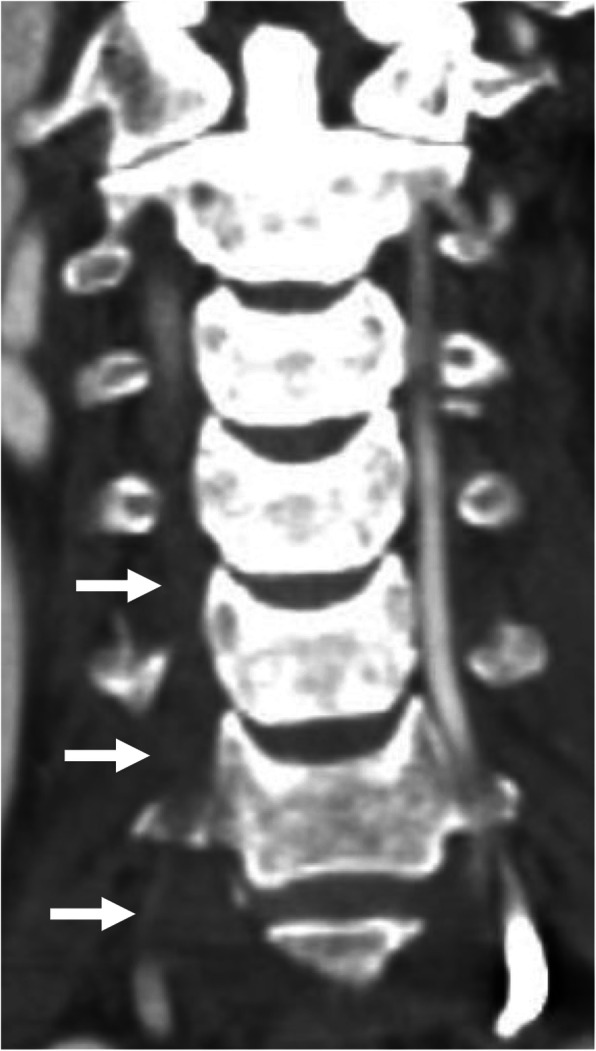
Coronal image of computed tomography angiography revealed a right blunt vertebral artery injury (arrows)

**Fig. 3:**
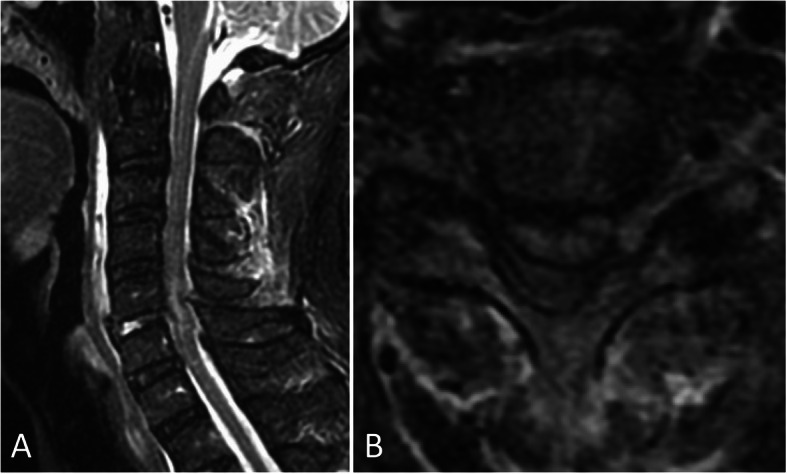
Magnetic resonance imaging using **a** sagittal short T1 inversion recovery (STIR) sequence and **b** axial T2 weighted image at C5/6 at re-admission shows disc injury and spinal cord compression by a posterior epidural mass accompanied by an intramedullary signal intensity change at this level. Prevertebral soft-tissue edema, injury of the interspinous ligament, and a narrowed canal also are evident. There is no flow void in right vertebral artery on axial T2 weighted image, suggesting vertebral artery injury

**Fig. 4:**
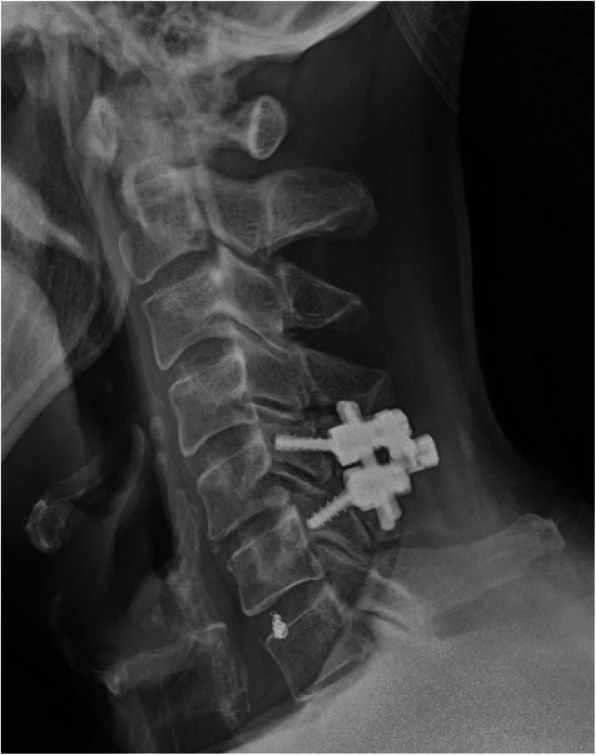
Postoperative lateral radiograph of the cervical spine. Lateral mass screws were inserted into C5 and C6 bilaterally. A coil for the vertebral artery injury at C6/7 also can be observed

An 81-year-old Asian man who was hit by a car while riding on a bike was transferred to our emergency room. He had no neurological symptoms except numbness in the right C6 dermatome area. Computed tomography (CT) of his cervical spine showed a right minimally displaced facet fracture of C6. The fracture fragment size was 6 mm, involving 25% of the height of the intact lateral mass (Fig. 1). CT angiography (CTA) at initial survey revealed a right blunt VAI, which was overlooked at initial admission (Fig. 2). CTA was used because it is integrated into a whole-body CT protocol for patients with high energy or multiple traumas. His blood data showed no abnormal findings of coagulation or platelet count. The fracture fragment was small, and the fracture was considered stable and the patient was treated with a Philadelphia collar and discharged four days after admission. We ordered magnetic resonance imaging (MRI); however, the patient could not undergo MRI during his first hospital stay. In addition to the MRI reservation being full, an MRI was deemed less urgent as he had no motor deficit. A visit to the outpatient department, including an MRI, was scheduled nine days after injury. However, 9 days after the injury, he developed quadriplegia gradually and was re-admitted to the hospital. He presented with complete paralysis of the lower extremity and bilateral motor weakness of the upper extremity including the elbow flexors and extensors, wrist extensors, and the finger abductors and flexors. Muscular power was graded as 0/5 to 4/5 by manual muscle testing (MMT). His neurological level of injury (NLI) was C4, and American Spinal Injury Association (ASIA) Impairment Scale was A. MRI at re-admission showed a disc injury at C5/6 with spinal cord compression from a posterior epidural mass accompanied by intramedullary signal intensity changes at the same level (Fig. 3). Immediately after admission we surgically performed a mid-splitting laminoplasty with instrumented fusion of C5–6 using lateral mass screws (Fig. 4). At surgery, fibrous scar tissue compressing the posterior aspect of the cord was observed and removed. We consulted a neurosurgeon about the blunt VAI, and the patient underwent endovascular stenting. At four months after surgery motor function in his upper extremities had improved and he was able to eat by himself using an assistive device but motor loss persisted in both of his legs.

### Case 2

**Fig. 5:**
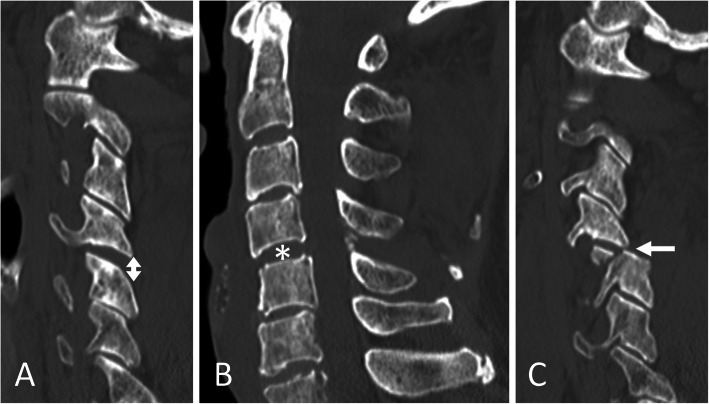
**a** Right parasagittal **b** midsagittal and **c** left parasagittal computed tomography scan of the cervical spine showed a widening of the right C4/5 facet joint space (double-headed arrow), widening of the disc space (asterisk) and the left facet fracture of C4/5 and anterior subluxation of C4 (arrow). The calcification of the yellow ligament at C4/5 and narrowed canal also are evident

**Fig. 6:**
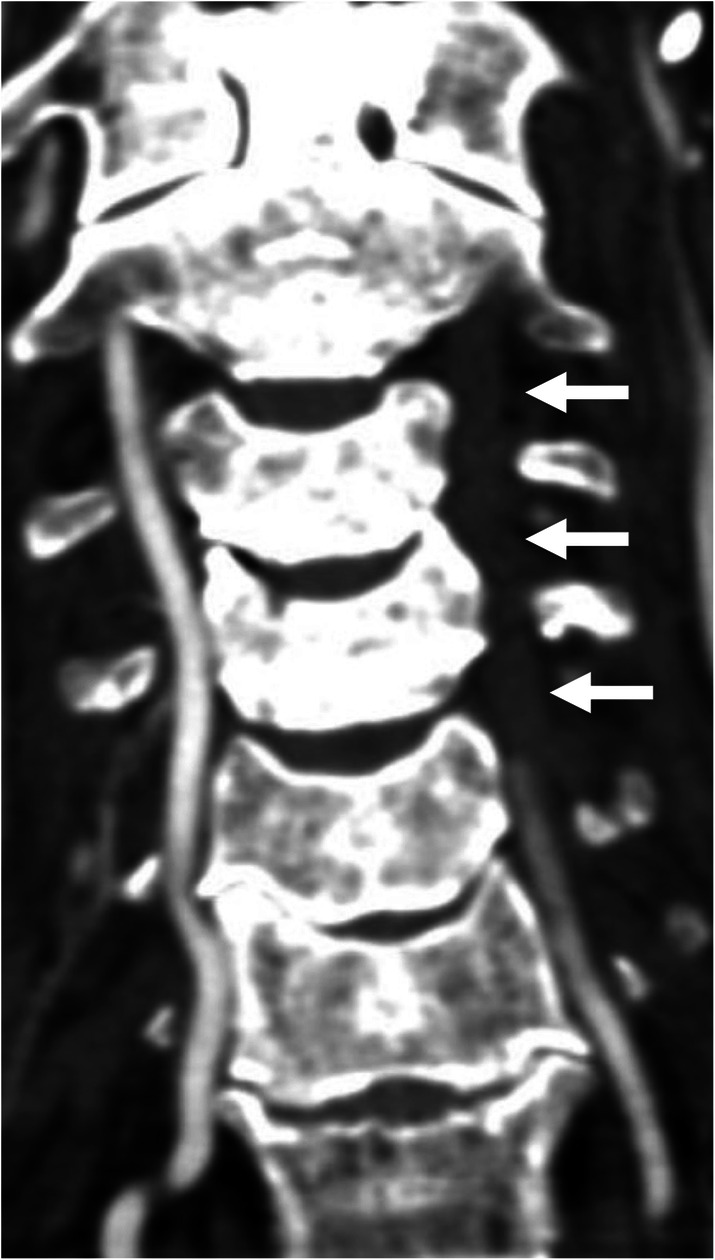
Computed tomography angiography in the coronal plane revealed a left blunt vertebral artery injury (arrows)

**Fig. 7:**
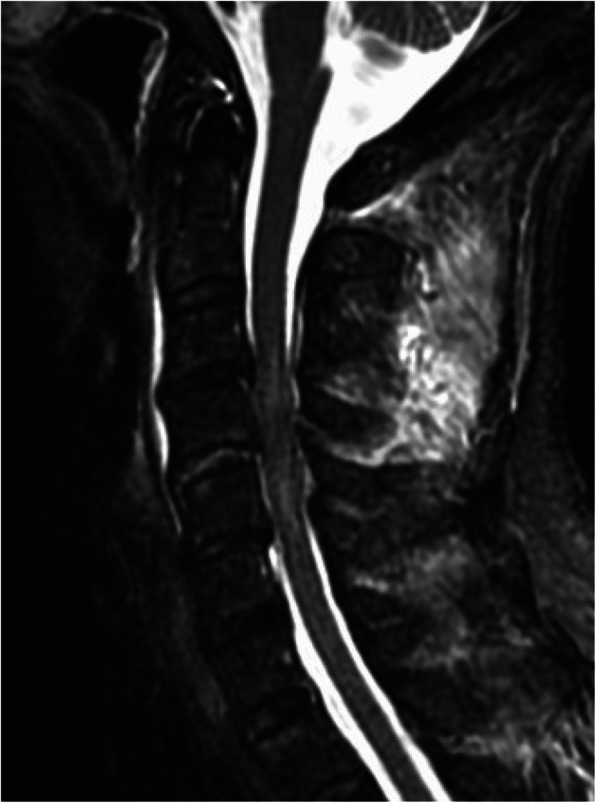
Magnetic resonance imaging using sagittal short T1 inversion recovery (STIR) sequence showed an intramedullary signal change at C4/5 with disc injury. Prevertebral soft-tissue edema and injury of the interspinous ligament, and narrowed canal also are evident

**Fig. 8:**
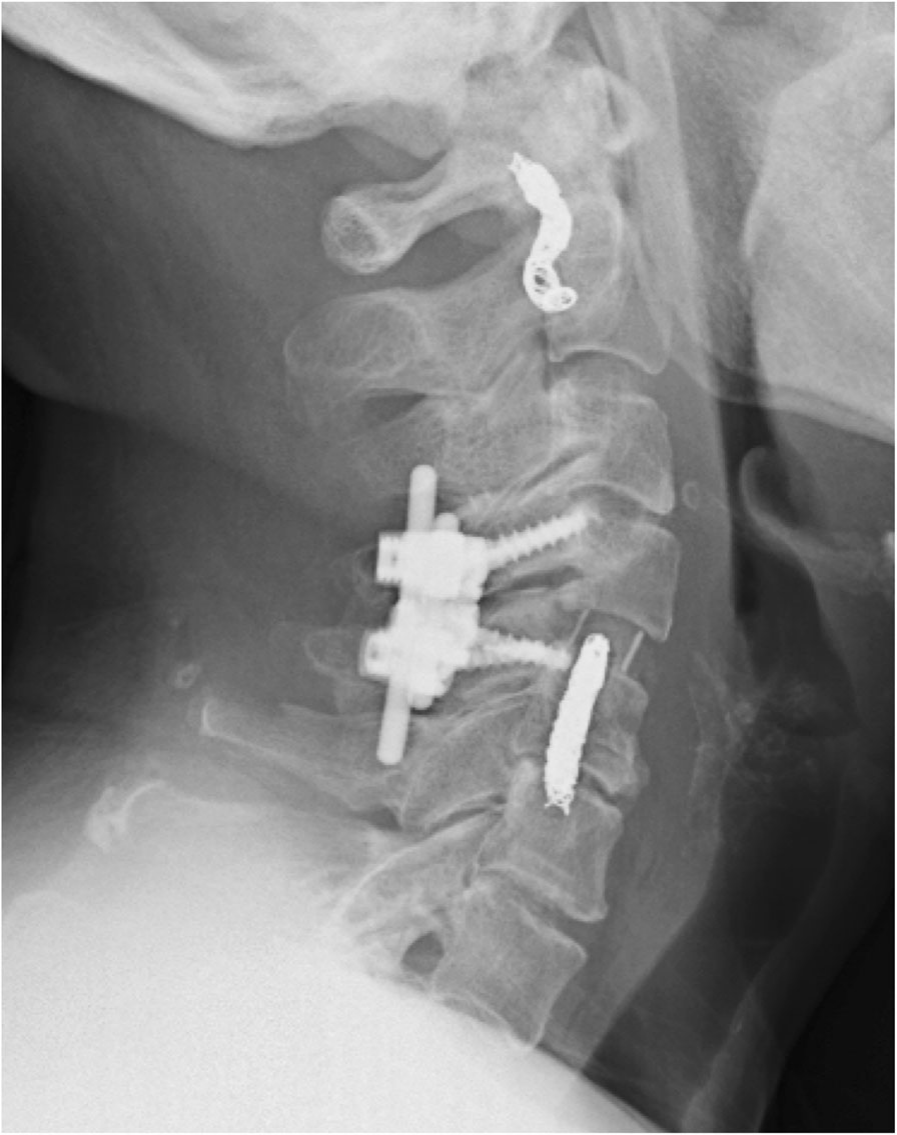
Postoperative lateral radiograph of the cervical spine. Lateral mass screws were inserted in C4 and C5 bilaterally. Coils for the vertebral artery injury at the C2 and C5 levels also can be seen

A 74-year-old Asian female who fell off a chair and hit her head was transferred to our emergency room. Upon arrival at our hospital, she had motor weakness of the left elbow flexors and extensors, wrist extensors, and both finger abductors. Muscular power was graded as 3/5 to 4/5 by MMT. There was no lower extremity weakness, indicating that she had central cord syndrome. Computed tomography of her cervical spine showed signs of instability, including a widening of the right C4/5 facet joint space, left facet fracture of C4/5 and anterior subluxation of C4 (Fig. 5). The fracture fragment size was 6 mm and involved 25% of the height of the intact lateral mass. An MRI showed an intramedullary signal change of the spinal cord with disc injury at the C4/5 level (Fig. 6). Preoperative CT angiography revealed a left blunt VAI (Fig. 7). We consulted a neurosurgeon, and the patient underwent endovascular stenting. After endovascular therapy, we performed combined anterior-posterior fusion surgery with a cage and lateral mass screws bilaterally at the C4–5 level (Fig. 8). For this case we used a combined anterior-posterior approach to avoid disc collapse and kyphosis. Generally, an anterior or posterior only approach yields favorable outcomes for this type of injury [4–8]. The patient presented numbness of her left arm but no motor weakness two years after surgery.

## Discussion and conclusion

A minimally displaced unilateral facet fracture can result in severe neurological compromise. From our experience with two cases, both disc injury and VAI are considered possible indicators for significant instability.

Our first patient was the first to be reported who developed quadriplegia a few days after injury during non-operative treatment of the unilateral facet fracture. Based on a previous study, failure of non-operative treatment dictated surgery to prevent listhesis progression or worsening of radiculopathy [4–6]. In that study, no patients were found to have new-onset myelopathy or catastrophic spinal cord related symptoms at the follow-up. Most of the patients in the prior studies initially were treated non-operatively, and 2 to 14 weeks later treated surgically if needed [4].

Several studies sought to define predictors of instability or failure of non-operative treatment in isolated cervical spine facet fractures. Van Eck et al. defined risk factors as the presence of radiculopathy at the time of presentation, a higher body mass index, increased Injury Severity Score (ISS), greater initial fracture displacement, and more than 2 mm of listhesis. Spector et al. found that patients with fractures involving more than 40% of the height of the intact lateral mass or an absolute height of more than 1 cm are at increased risk for failure of nonoperative treatment [6]. Aarabi et al. could not find any correlation between instability and any of the predictors, including conventional demographic, clinical, imaging, or injury severity variables, morphology classifications, or instability checklists [5]. Most studies on this topic have used a study design in which the outcomes of nonoperative treatment were compared to operative treatment. This design leads to a high degree of selection bias. Among proposed risk factors, our first patient only had radiculopathy at his initial presentation. Risk factors were not applicable to our second patient because she presented with central cord syndrome immediately after injury, although she had a similar spinal injury pattern as the first patient.

Halliday et al. recommended selecting surgical treatment based on the presence of a subluxation and the integrity of the ligamentous structures [9]. Following MRI evaluation, the anatomical integrity of the anterior and posterior longitudinal ligaments, the facet capsule, and the interspinous ligament were analyzed. They concluded that surgical intervention was indicated if 3 of 4 ligaments were damaged after trauma. A biomechanical study also showed that partial injury to the intervertebral disc resulted in a significant increase in angular displacement [10] whereas superior articular facet fractures alone involving 40% of the lateral mass did not necessarily result in intervertebral instability. CT was the mainstay in diagnosis and decision-making in most prior studies for this type of fracture, and MRI was used in only a limited number of patients. However, we recommend that patients should undergo MRI to assess anterior soft tissue injury if there is any evidence of bone injury even without neurologic deficit because of higher sensitivity of MRI for detection of acute soft tissue injury compared to CT [11]. Dynamic flexion/extension radiographs might allow an imaging evaluation for this patient population. However, dynamic flexion/extension radiographs remain a level 3 recommendation in the guidelines for the management of acute cervical spine and spinal cord injuries [12] because these radiographs tend to fail to identify the ligamentous injuries identified on MRI [13, 14].

VAI associated with cervical spine injury is a marker for more severely injured patients [15]. Facet fractures without dislocation account for only 6% of the VAIs associated with a cervical spine injury [16]. Higher energy injury mechanisms may result in fracture dislocation accompanied by VAI [16]. Facet fracture without dislocation has been reported to be relatively stable and to yield favorable outcomes when compared to other types of cervical spine fracture. Facet fracture without dislocation is the result of hyperextension, lateral compression, and rotation of the cervical spine, and has been classified by Allen et al. as a “compression-extension Stage 1” fracture [7, 8, 17].

This is the first report of an isolated unilateral facet fracture that resulted in catastrophic spinal cord injury. These two cases illustrate that an isolated minimally displaced unilateral facet fracture with disc injury and VAI were associated with significant instability that can lead to spinal cord injury. Clinicians should be aware of disc injury and VAI as possible signs of intervertebral instability in patients with an isolated unilateral facet fracture.

## Data Availability

Data sharing is not applicable to this article as no datasets were generated or analyzed during the current study.

## Abbreviations

VAI: Vertebral artery injury
CT: Computed tomography
CTA: Computed tomography angiography
MRI: Magnetic resonance imaging
MMT: Manual muscle testing
NLI: Neurological level of injury
ASIA: American Spinal Injury Association
ISS: Injury Severity Score
